# Epi-revolution in rheumatology: the potential of histone deacetylase inhibitors for targeted rheumatoid arthritis intervention

**DOI:** 10.1007/s10787-024-01486-z

**Published:** 2024-05-07

**Authors:** Padmini Pai, Aradhika Vijeev, Sharada Phadke, Manasa Gangadhar Shetty, Babitha Kampa Sundara

**Affiliations:** 1 Department of Biophysics, Manipal School of Life Sciences, Manipal Academy of Higher Education, Manipal, 576104 Karnataka India; 2 Manipal School of Life Sciences, Manipal Academy of Higher Education, Manipal, Karnataka India

**Keywords:** Rheumatoid arthritis, Inflammatory cytokines, Fibroblast-like synoviocytes, Histone deacetylase, Matrix metalloproteinase

## Abstract

Autoimmune diseases hold significant importance in the realm of medical research, prompting a thorough exploration of potential therapeutic interventions. One crucial aspect of this exploration involves understanding the intricate processes of histone acetylation and deacetylation. Histone acetylation, facilitated by histone acetyl transferases (HATs), is instrumental in rendering DNA transcriptionally active. Conversely, histone deacetylases (HDACs) are responsible for the removal of acetyl groups, influencing gene expression regulation. The upregulation of HDACs, observed in various cancers, has steered attention towards histone deacetylase inhibitors (HDACi) as promising anti-cancer agents. Beyond cancer, HDACi has demonstrated anti-inflammatory properties, prompting interest in their potential therapeutic applications for inflammatory diseases such as rheumatoid arthritis (RA). RA, characterized by the immune system erroneously attacking healthy cells, leads to joint inflammation. Recent studies suggest that HDACi could offer a viable therapeutic strategy for RA, with potential mechanisms including the inhibition of synovial tissue growth and suppression of pro-inflammatory cytokines. Furthermore, HDACi may exert protective effects on bone and cartilage, common targets in RA pathology. In-depth investigations through in vivo and histopathology studies contribute to the ongoing discourse on the therapeutic benefits of HDACis in the context of RA treatment.

**Graphical Abstract:**

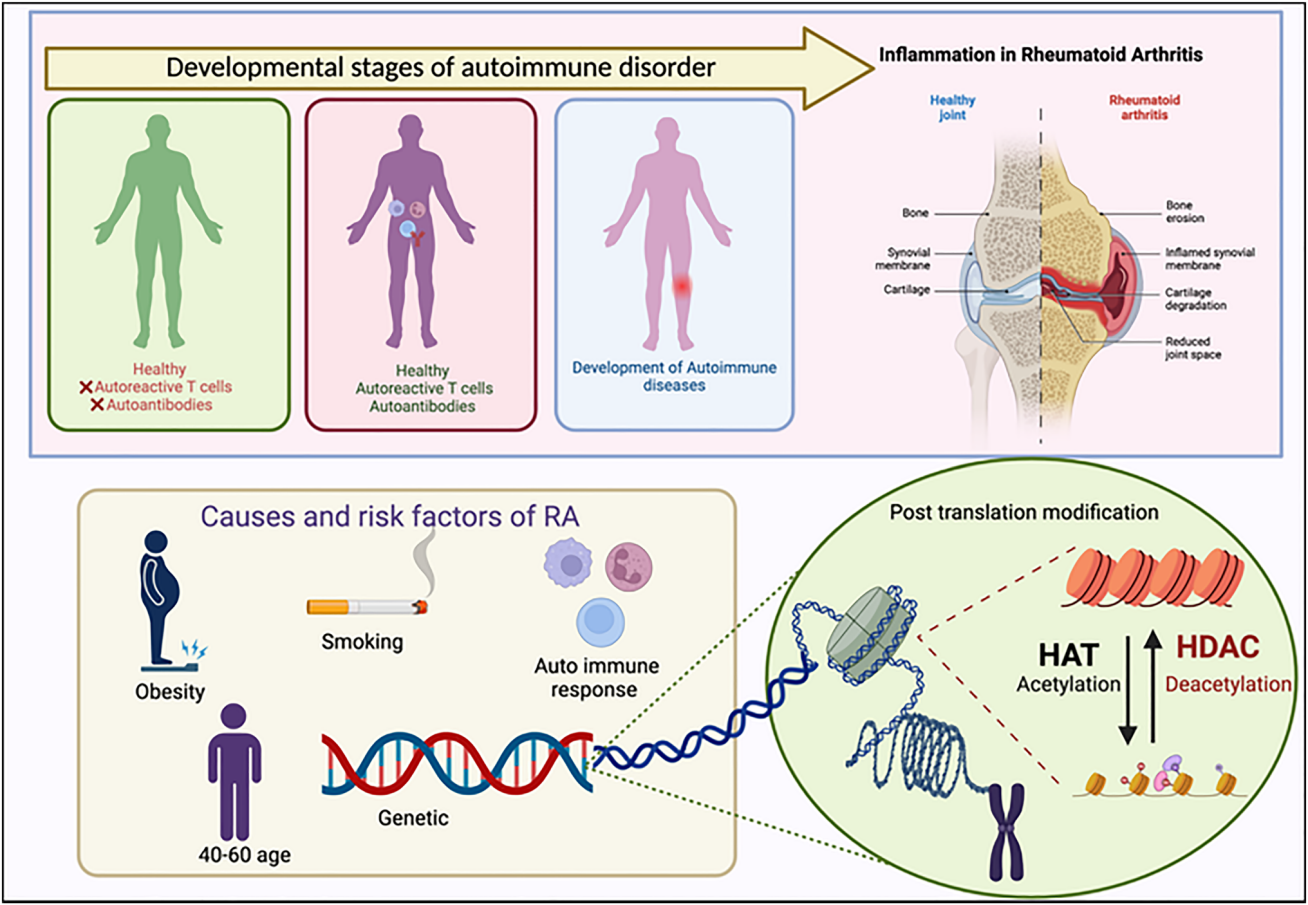

## Introduction

**Fig. 1:**
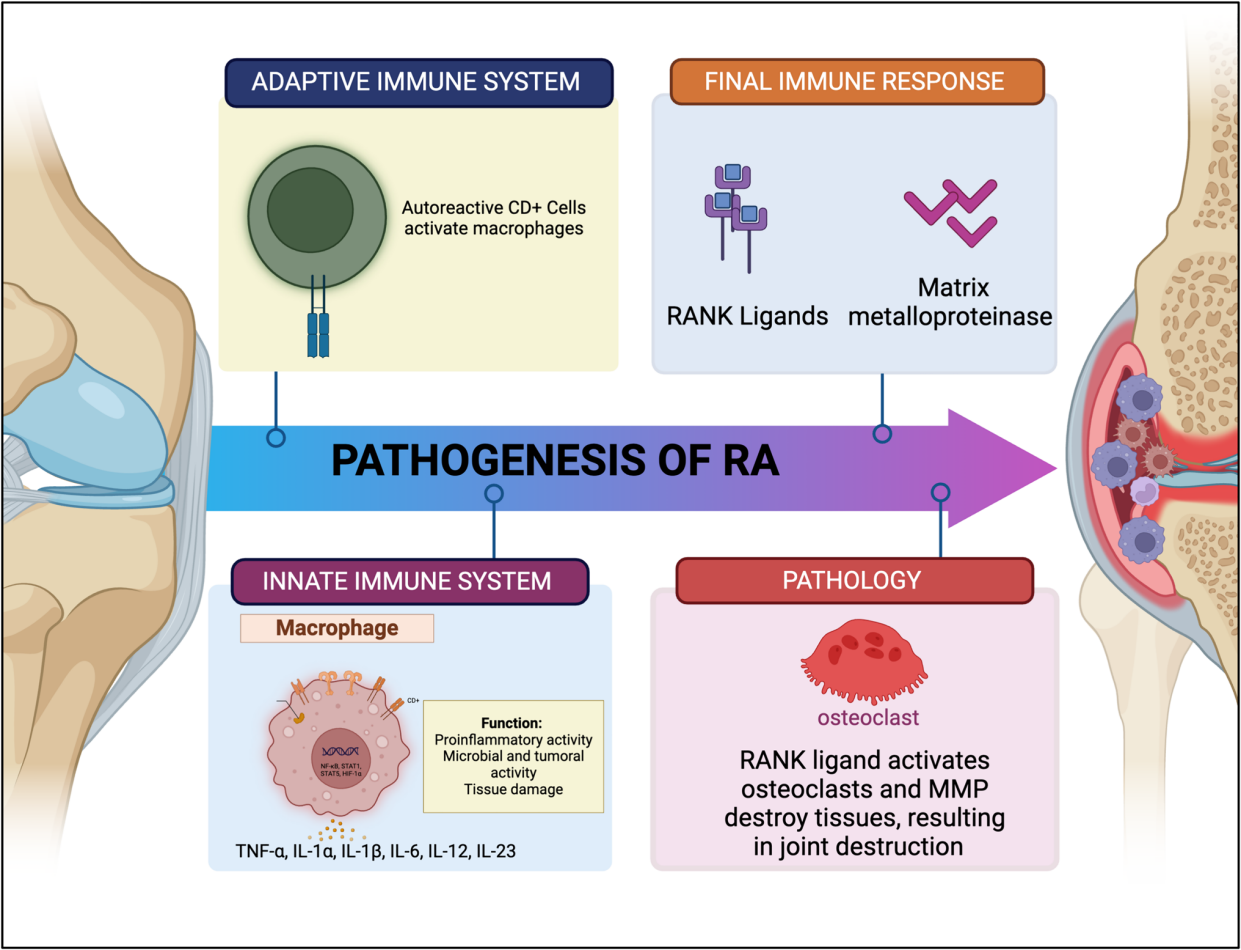
Pathogenesis of rheumatoid arthritis

Rheumatoid arthritis (RA) falls under the classification of autoimmune diseases, marked by inflammation that predominantly targets joints, and the presence of synovial swelling. Prior research has indicated a significant association between sex hormones and the pathogenesis of RA, with women exhibiting a higher susceptibility to the disease than men, ranging from 2 to 3 times more likely (Gerosa et al. 2008). This condition is characterized by systemic participation, affecting various organs, which may encompass, among others, the lungs, kidneys, and joints (Wu et al. 2022). An illustrative instance is the presence of RA associated Interstitial Lung Disease (RA-ILD) (Yuan et al. 2020). RA is classified as an autoimmune condition, wherein the body's cells are targeted, and an immune reaction is triggered against the molecules within one's body (Smith et al., 1999). The aetiology of RA initiates within the synovial membrane, encompassing the afflicted joints and then illness advances from synovia to cartilage (Burrage et al. 2006). Recruiting activated effector cells involved in the immune response, such as, B-lymphocytes, and T-lymphocytes to the joints impacted by RA has an essential role in mediating its pathogenesis (Thalhamer et al. 2008). These activated immune cells are known to infiltrate the synovial membrane and induce alterations in the sub-lining of the synovium (Lee and Weinblatt 2001; Mulherin et al. 1996). Various cytokines are released by these cells, including interleukin (IL) IL-1, IL-2, IL-4, IL-6, IL-8, IL-10, IL-17, IL-18, tumor necrosis factor-alpha (TNF-α) and RANK ligands which activate synovial fibroblasts, which in turn release more inflammatory cytokines and enzymes responsible for cartilage degradation (Grabiec et al., 2011). These cytokines trigger the initiation of diverse mechanisms that play a part in the development of RA, including essential pathogenesis events such as the increase in the cell population of RA synovial fibroblasts (RASFs), the generation of enzymes that breakdown bone and cartilage, colony-forming factors including granulocyte macrophage colony-stimulating factor (GM-CSF), and production of cytokines such as IL-8 (Sweeney and Firestein 2004) (Fig. 1).

In addition to cytokines, several other factors, such as pro-angiogenic factors, CAMs (Cell Adhesion Molecule) and MMPs (Matrix Metalloproteinase) have vital contributions to the progression of diseases (Lee and Weinblatt 2001). MMPs are enzymes that have a vital role in cell migration, angiogenesis and bone degradation with respect to RA. The synovial cells and macrophages are responsible for producing pro-angiogenic substances essential for providing nutrition to the circulating leukocytes, which contribute to the development and maintenance of hypertrophic joints (Elshabrawy et al. 2015). The NF-κB pathway, which is also affected by HDAC activity, is also a pivotal participant in the process of inflammation. It regulates the transcription of genes that have a significant impact on the development of RA such as MMPs, and inflammatory cytokines (Burrage et al. 2006; Hawtree et al. 2013). Epigenetic factors have a fundamental role in governing gene expression (Costenbader et al. 2012). One example of an epigenetic alteration is acetylation, which is facilitated by a cluster of enzymes known as HDACs and HATs (Ruijter et al. 2003). HDACis have shown to reduce expression of inflammatory genes by mechanisms such as inhibition of mRNA stabilities like that of IL-6 (Grabiec et al. 2011).

Studies conducted over the past 2 decades have yielded favorable outcomes regarding the effect of HDACi therapies on the progress of RA (Klein & Gay 2013; Kong et al. 2013). Inhibition of HDAC activity has shown to suppress inflammatory cytokine production in RA (Angiolilli et al. 2014).

**Fig. 2:**
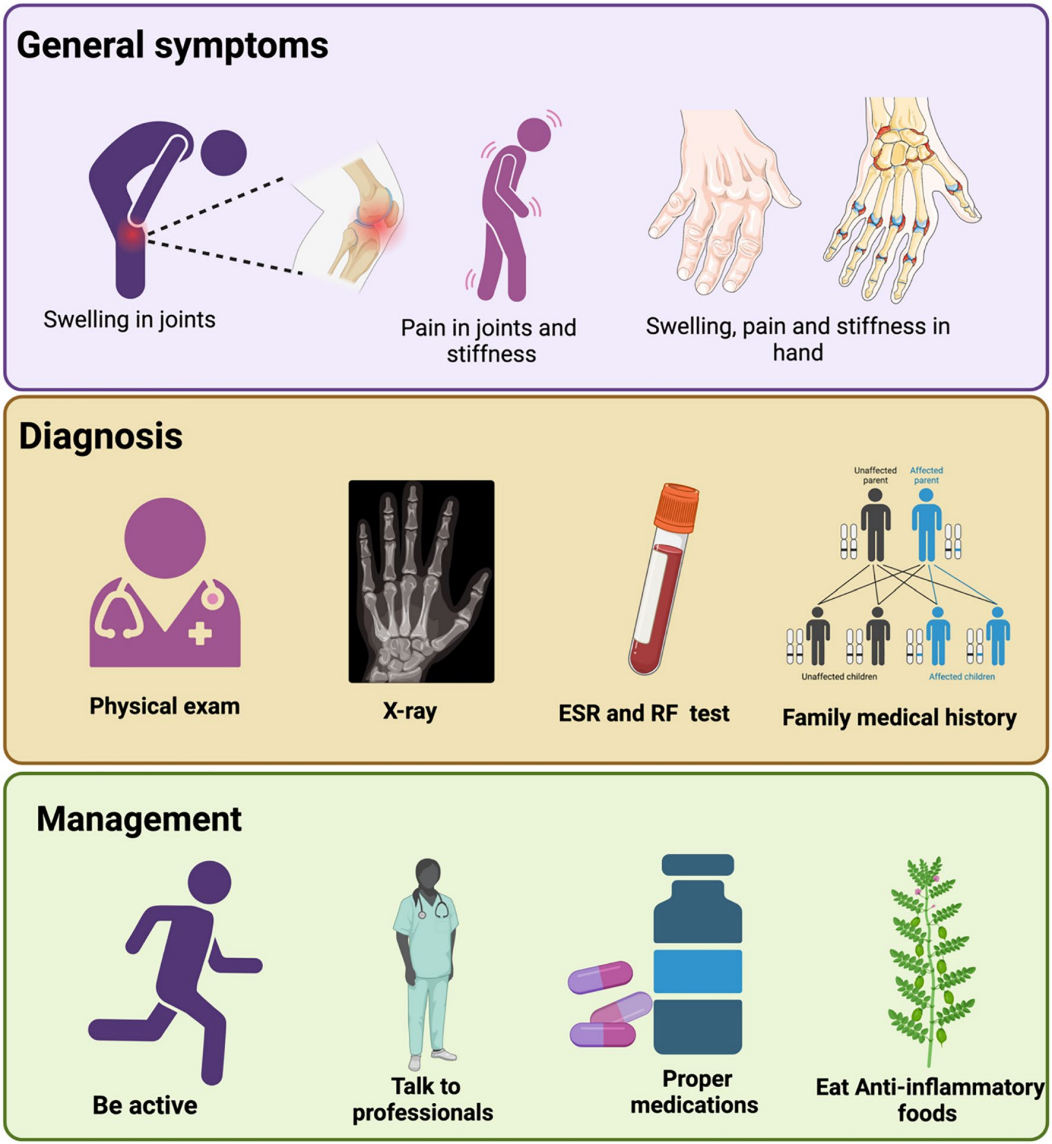
General symptoms, diagnosis, and management measures of RA

Furthermore, HDAC inhibitors have been investigated for their potential as therapeutic agents in clinical trials for RA patients, although more research is needed to fully elucidate their efficacy and safety profiles in this context. In terms of diagnosis, while there are no specific HDAC-based diagnostic tests for RA, advancements in biomarker research, such as the identification of epigenetic modifications associated with RA, hold promise for improving early detection and personalized treatment strategies for individuals with this debilitating condition. Diagnosing and treating RA presents a complex set of challenges for healthcare professionals. The diagnosis process can be facilitated by using magnetic resonance imaging (MRI) and biopsy analysis. In addition to this, radiographic evidence can be obtained, which often helps diagnosis before actual physical dysfunction sets in (Lee & Weinblatt 2001). Diagnosis often involves a combination of clinical evaluation, laboratory tests, and imaging studies to assess joint inflammation, swelling, and the presence of specific antibodies like rheumatoid factor and anti-cyclic citrullinated peptide (Fig. 2).

Early and accurate diagnosis is crucial to initiate timely intervention and prevent irreversible joint damage. Treatment strategies for RA typically encompass a multidisciplinary approach, including pharmacological interventions, physical therapy, and lifestyle modifications. DMARDs, such as methotrexate, are commonly prescribed to suppress inflammation and slow down joint damage (O’dell et al., 2013). Biologic agents and targeted synthetic DMARDs provide additional options for those who do not respond adequately to traditional therapies. Despite significant advancements in RA management, challenges persist. Achieving optimal disease control and managing symptoms can be elusive, requiring ongoing adjustments to treatment plans. Moreover, the potential for adverse effects associated with long-term medication use and the varying response among individuals contribute to the complexity of RA management. Additionally, access to specialized care, the high cost of some medications, and the impact of RA on daily functioning pose additional hurdles. Research continues to address these challenges, striving to enhance diagnostic precision, optimize treatment protocols, and improve the overall quality of life for individuals living with rheumatoid arthritis.

This review specifically addresses the involvement of HDACs in RA and elucidates the epigenetic mechanisms that could contribute to the pathogenesis of the disease. Research indicates that disturbances in HDAC activity may impact the abnormal gene expression observed in rheumatoid arthritis. A deeper understanding of the intricate interplay between HDACs and RA has the potential to reveal therapeutic targets and innovative treatment approaches for this intricate autoimmune condition. The review incorporates a segment discussing the correlation between HDAC and RA. Additionally, it highlights the impact of HDACis on RA. The comprehensive review of existing literature on the association between RA and HDACs offers valuable insights into the molecular mechanisms at the core of RA, paving the way for the exploration and development of targeted therapies.

## Role of HDAC in RA pathogenesis

RA has an immensely coordinated pathophysiology, which involves various processes like synovial hyperplasia, pannus formation, bone erosion, synovial cell infiltration in joints, and cartilage destruction. However, new studies have indicated that epigenetics have a vital role in the immune response observed in RA (Vijaykrishnaraj et al. 2023). Alterations in the expression and functioning of DNA and histone-modifying regulatory proteins are instrumental in the pathology of RA. Compelling preclinical reports from recent times have demonstrated that targeting these proteins, especially the ones associated with acetylation, such as HDACs, may give rise to new therapeutic avenues in RA (Grabiec and Reedquist 2013).

Compared to patients with OA and healthy controls, it was seen that the synovial tissue of RA patients had significantly lower levels of overall HDAC activity. Additionally, it was noted that there was no different activity with respect to HAT. Histone activity shifted towards hyperacetylation in RA patients, as determined by calculating the ratio of HDAC to HAT. This suggested a significant association between decreased HDAC activity and chronic inflammatory processes. The observation that the synovium of healthy individuals possessed higher levels of HDAC activity served as more evidence for this hypothesis. It was determined that an essential factor in the pathophysiology of RA was, the observed decrease in class I HDAC activity, most likely due to the activation of proinflammatory transcription factors (Huber et al. 2007). HDAC1 is overexpressed in rheumatoid arthritis synovial fibroblast (RA-SF). HDAC1 inhibits MMP-1 synthesis but promotes RA-SF cell survival and proliferation. RA-SF is characterized by HDAC1 overexpression. HDAC1 has two distinct roles in RA-SF it contributes to cell proliferation, while suppressing MMP-1 production. Additionally, HDAC2 is crucial for RA-SF proliferation and apoptosis. Inhibiting HDAC2 may be a sensible tactic to decrease RA-SF proliferation without altering MMP production, as HDAC1 and HDAC2 have comparable effects on RA-SF proliferation (Horiuchi et al. 2009).

Nuclear HDAC activity was associated with the level of cytoplasmic TNF-α and was considerably higher in RA compared to OA and normal controls. In comparison to OA and normal controls, RA synovial tissue had greater levels of HDAC1 mRNA expression, which correlated positively with TNFα mRNA expression. Nuclear HDAC1 protein levels were found to be raised in RA, when contrasted with the synovial tissue of OA. TNF-α stimulation markedly elevated the nuclear HDAC activity and HDAC1 mRNA expression at 24 h and HDAC1 protein expression at 48 h in RASFs (Kawabata et al. 2010). It was observed that the levels of HAT and HDAC activity in RA and healthy controls were comparable. While Sirt drastically decreased HAT in RA, ex vivo treatment of peripheral blood mononuclear cells (PBMC) by HDACi tended to lower HDAC expression in healthy controls. HDACi could influence the expression of HDAC and HAT PBMC, particularly Sirt in RA. The imbalanced pattern of HAT and HDAC activity could potentially explain why HDACi could be used as a therapy for inflammatory rheumatic conditions (Toussirot et al. 2013). A portion of type I interferon response genes are transcriptionally transcribed and IRF1's nuclear localization is promoted by the suppression of RA FLS HDAC5 expression by inflammatory cytokines. The results of the study reveal HDAC5 as a novel inflammatory mediator in RA and indicate that methods for restoring HDAC5 expression in vivo or the creation of HDAC inhibitors that do not interfere with HDAC5 function, may prove beneficial in the treatment of RA (Angiolilli et al. 2014). HDAC1 expression in RA SFs was much higher than in osteo arthritis synovial fibroblasts. In vitro, HDAC1^KD^ led to diminished proliferation, invasion, and migration and transcriptome profiling further demonstrated effects on the expression of genes mediating inflammation, invasion, and proliferation. Moreover, HDAC1 inhibition in collagen induced arthritis (CIA) produced decreased TNF in joint tissue, decreased bone and cartilage degradation, and lesser joint swelling. These findings support the potential therapeutic efficacy of HDAC1 inhibitors by identifying it as an important mediator of tissue destruction in RA (Hawtree et al. 2015). It was observed that altered STAT expression and function are a requirement for the anti-inflammatory actions of HDAC3 depletion in macrophages. The effects of pan-HDACi in inhibiting inflammatory gene expression, including type I IFN production in RA FLS, were largely reconstructed by inhibiting HDAC3 in RA FLS. Evidence suggesting HDAC3 may serve as a vital epigenetic regulator of inflammation were presented by this study (Angiolilli et al. 2017).

**Fig. 3:**
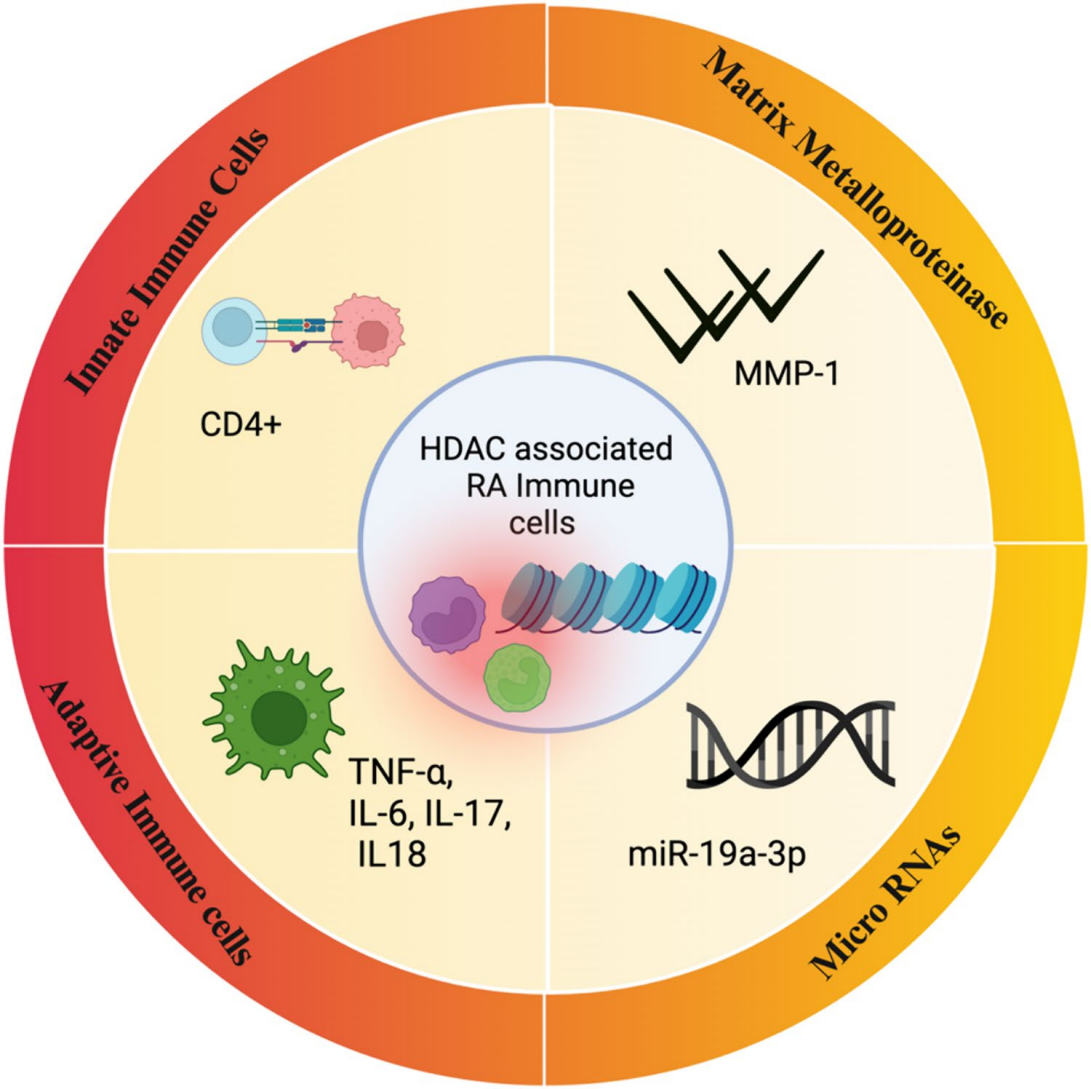
HDAC-associated RA immune cells

In contrast to healthy controls, PBMCs from RA patients had a reduced amount of class I HDAC expression in terms of both mRNA and protein. There was a significant increase in nuclear HAT activity. Furthermore, it was discovered that among all the HDACs lowered overall, HDAC3 activity was the most dramatically reduced within the RA group. When compared to healthy controls, PBMCs from RA patients had higher levels of total histone H3 acetylation, but not H4 acetylation. Class I HDAC expression and activity are downregulated in RA PBMCs, while HAT activity is upregulated. In RA PBMCs, there was an imbalance in the degree of HDAC and HAT activity (Li et al. 2018). CIA is unable to affect mice with a T cell-specific deletion of HDAC1 (HDAC1-cKO). On the contrary, the antibody response to collagen type II remains unaltered, implying that T cell-mediated B cell activation remains unaltered. The serum of HDAC1-cKO mice showed a substantial decrease in the inflammatory cytokines IL-6 and IL-17. HDAC1-deficient CD4 + T cells treated with IL-6 demonstrated a reduced expression of CCR6. These findings point to a critical role for HDAC1 in the pathophysiology of CIA and raise the prospect that selective HDAC inhibitors (HDACi) could be effective in targeting HDAC and other class I HDACs, to treat RA (Göschl et al., 2019). In individuals with RA and mice models, HDAC3 and IL17RA were over-expressed, whereas miR-19a-3p was under-expressed. The target gene of miR-19a-3p is IL17RA, and miR-19a-3p may decrease RA-ILD by negatively regulating IL17RA. In conclusion, HDAC3 increased the expression of IL17RA mediated by miR-19a-3p, which in turn promoted the development of RA-ILD fibrosis (Fig. 3) (Yuan et al. 2020).

## HDACi as therapeutic agents in RA

HDAC inhibitors fall under synthetic molecules, chemical derivatives or even naturally occurring compounds which can be used as potential therapeutic agents. These chemicals inhibit HDACs and thereby influence RA pathogenesis, as discussed in this review (Vijayakrishnaraj et al., 2023).

HDACis affect cell differentiation and proliferation (Lee et al. 2020). Some inhibitors known to tone down the symptoms of inflammatory conditions, such as paw swelling, clinical arthritis score, bone erosion, and cartilage destruction are Trichostatin A (TSA), Suberoylanilide hydroxamic acid (SAHA) (Vijaykrishnaraj et al. 2023; Lee et al. 2020).

### MS-275

MS-275, a HDACi alternatively referred to as Entinostat and SNDX-275, is currently undergoing clinical trials aimed at suppressing lymphoma, solid tumors, and leukemia through HDAC-mediated therapies (Lee et al. 2020). The 2007 study by Lin and colleagues found that the administration of MS-275 demonstrated a considerable reduction in paw swelling and induced a decrease in bone degeneration, at lower dosages. RA-associated bone loss and resorption were notably reduced (Lin et al. 2007). The efficiency and effectiveness of MS-275 as an inhibitor in CIA models has been demonstrated by Hawtree and colleagues in 2015, as indicated by its preferential targeting of class 1 HDACs, with a specific emphasis on HDAC1. The suppression of HDAC1 led to a marked reduction in the production of TNF in the joints affected by inflammation in rat models of CIA. There was a reduced extent of bone and cartilage degradation at the histology level (Hawtree et al. 2015). CCR6 is an important chemokine receptor for the development of CIA. The class 1 HDAC inhibitor, MS-275, was shown to reduce CCR6 (chemokine receptor 6) expression levels in CD4 + T cells (Göschl et al. 2020).

### Trichostatin A (TSA)

Trichostatin A (TSA) falls within the isohydroxamic acid classification, and it has been observed to exhibit properties such as neuroprotection and anti-inflammation (Cui et al. 2019; Lv et al. 2021). An early study displaying the therapeutic effects of TSA is one by Chung and colleagues. The 2003 study focused on the effect of Phenylbutyrate (PBA) and TSA on RA development, and they were observed to suppress TNF-α expression in affected tissues in the synovium while simultaneously promoting p21^Cip1^ and p16^INK4^ expression in cells of the synovial tissue during adjuvant arthritis, thus decreasing joint swelling and bone and cartilage degradation, among other symptoms being alleviated (Chung et al. 2003). Morinobu and colleagues in 2006 saw that TSA decreased the growth of RA-SF cells depending on the dosage administered. Additionally, it increased the expression of p21^WAF1/CIP1^, a known cell cycle inhibitor (Morinobu et al. 2006). Additionally, along with TSA, combination with Ultrasound (US) therapy, as studied by Nakamura and colleagues in 2008, led to a notable reduction in the cell population in the S phase of the cell cycle while upregulating the cell population in the G2-M phase of the cell cycle. Combined therapy yielded significant reductions in cellular viability and notably induced apoptosis in RASFs with high efficiency (Nakamura et al. 2008).

A more in-depth study outlining the mechanisms followed by TSA was done by Zhang and colleagues in 2016. After exposing RA-FLSs to a 24-h treatment with TSA, it was evident that their invasive capacity was significantly hindered, particularly under hypoxic conditions. Treatment with TSA effectively inhibited the upregulation of MMP-2 and MMP-9 in hypoxic conditions. With respect to the cell cycle, the link between the PI3K/Akt signaling pathway and cancer cell proliferation and invasive characteristics is well established. In the context of hypoxic RA-FLSs, TSA demonstrated its ability to suppress the PI3K/Akt signaling pathway. These results and therapeutic mechanisms reveal insights into the potential therapeutic applications of TSA in relation to RA (Zhang and Zhang 2016). Grabiec et al. 2010 studied that both TSA and Nicotinamide (NIC) have been observed to selectively target and decrease the expression and upregulation of Bfl-1, an antiapoptotic member of the Bcl-2 family, in macrophages and RASFs. As a result, these compounds induce apoptosis in these specific cell types. Both reduced LPS-induced TNF-a, and IL-6, in macrophages 2(Grabiec et al. 2010). Mizutani et al. 2010 showed that while both TSA and FK228 trigger apoptosis, only FK228 was observed to exert its effects through hydrogen peroxide (H_2_O_2_)-mediated pathways (Mizutani et al. 2010).

### Givinostat

ITF2357, also known as Givinostat, is an HDACi which at low concentrations, displays anti-inflammatory properties (Furlan et al. 2011) and downregulates gene expression where related to the JAK/STAT (Janus kinase/signal transducers and activators of transcription) signalling pathway (Savino et al. 2017). In a study conducted by Vojinovic et al., in 2011, Givinostat demonstrated a strong acceptance as well as a good safety record, with only a limited number of adverse events reported. A notable primary observation was reducing the prevalence of joints affected by active RA or experiencing a limited range of motion (Vojinovic and Damjanov 2011). Grabiec et al. in 2011 studied that both TSA and ITF2357 demonstrated the ability to inhibit the generation of IL6 produced by IL-1β, with RASF viability remaining unaffected. TSA specifically induced the suppression of IL-6 mRNA accumulation (Grabiec et al. 2011). In PBMCs obtained from both RA patients and healthy individuals, as supported by Gillespie and colleagues through their 2012 study, the production of key inflammatory cytokines, such as IFNγ, TNF, and IL-6, was notably suppressed by TSA and MI192 (HDAC3 selective inhibitor) individually (Gillespie et al. 2012). The administration of ITF2357 has been demonstrated to decrease the stability of mRNA transcripts encoding IL6, IL8, CXCL2, and PTGS2 in the study conducted by Angiolilli and colleagues in 2018 (Angiolilli et al. 2018).

### Tubastatin A

Tubastatin A is a selective inhibitor of HDAC6 (Shen et al. 2020). The administration of Tubastatin A had a dose-dependent effect in blocking the synthesis of pro-inflammatory cytokines, including TNF-α and IL-6, in THP-1 cells that were subjected to LPS and activated. Tubastatin A reduces pro-inflammatory chemokine secretion, such as Nitric Oxide (Vishwakarma et al. 2013). Oh et al. 2017 conducted a comparative analysis to assess the effects of CKD-L, which is a new HDAC6 inhibitor, compared to ITF2357 or Tubastatin A on the progression of RA. Notably, the administration of CKD-L and Tubastatin A led to a marked minimization when it came to the severity of RA in murine models. Notably, the impact of CKD-L and Tubastatin A on cell viability was found to be insignificant. In contrast, ITF-2357 decreased viability at concentrations exceeding 1 μM. Additionally, the TNF mRNA levels were dramatically reduced due to CKD-L treatment. TNF secretion was reduced by the administration of Tubastatin A and ITF2357, but the secretion of IL-1β or IL-10 remained unaltered (Oh et al. 2017).

### FK228

FK228, otherwise known as depsipeptide, is an HDACi showing prominent antitumor effects by inducing gene expression resulting in cell growth inhibition, apoptosis, induction of cell differentiation and angiogenesis inhibition (Konstantinopoulos et al. 2006; Mizutani et al. 2010). Few studies discussed in this review note the effects of FK228 on RA progression. In a 2004 study, Nishida and colleagues showed a dose-dependent suppression of RASF growth in vitro by FK228. This target is met by stimulating the production of p16^INK4a^ and the upregulation of p21^WAF1/Cip1^ synthesis in RASFs, ultimately ceasing the phase progression of the cell cycle (Nishida et al. 2004). FK228 shows inhibition in cell count increase, as seen in synovial sarcoma cells, in the study done by Ito and colleagues in 2005 (Ito et al. 2005). FK228 may offer therapeutic benefits for RA by inducing suppression of the synthesis of mRNA and protein expression of VEGF and HIF-1α (Hypoxia-Inducible Factor-1α), both of which possess pivotal roles in the angiogenesis process (Manabe et al. 2008).

### SAHA and MS-275

Lin et al. (2007) discovered that SAHA reduced inflammation associated paw swelling, diminished bone degradation in rats and mice and reduced effects of RA-induced bone resorption in rats (Lin et al. 2007). Choo et al. in 2010 studied MS275 and SAHA and found that the growth of human RA synovial fibroblastic E11 cells was observed to be significantly reduced by them in a non-cytotoxic manner. Reduced proliferation was associated with induction of the inhibitor of the cyclin-dependent kinase, p21, resulting cell cycle inhibition in the G0/G1 phase. Furthermore, the MS-275 and SAHA compounds decreased the production of pro-angiogenic factors, which include VEGF (Vascular endothelial growth factor), MMP-2, and MMP-9 in E11 cells at lower concentrations. The nuclear accumulation of NF-κB and p65, as well as the generation of IL-1b, IL-6, IL-18 and TNF-a, produced by LPS (Lipopolysaccharide) in THP-1 monocytic cells, were comparably reduced (Choo et al. 2010). Choo and colleagues 2013 showed that MS-275 and SAHA exerted their suppressive effects on the p38 MAPK signalling pathway, which plays a key role in chronic inflammation (Choo et al. 2013). As compared to SAHA, MS-275 exhibits greater efficacy in mitigating bone degradation and inflammation in CIA among rodents, in comparison to the pan-HDACi SAHA (Angiolilli et al. 2014). The HDACi BML-281, shows potential for HDAC6 inhibitors to be used as clinical treatments and for the prevention of conditions such as colonic inflammation in humans, through the promising results shown in studies (Choi et al. 2023). Lohman and colleagues in 2015 studied the anti-inflammatory effects of SAHA and BML281. The compounds had favourable effects in lowering the populations of pathogenic macrophages/osteoclasts and slowing disease development only at the low dose. At concentrations below 3 µM, these substances had anti-inflammatory properties on human macrophages. However, they exhibited pro-inflammatory actions at concentrations over 3 µM, which were still non-cytotoxic. Both compounds exhibited the ability to suppress the production of the cytokines IL12p40 and IL6, with efficacious doses observed up to 30 µM. Interestingly, it was observed that the production of TNF-α and IL1 was dramatically upregulated in a dose-dependent course when the concentrations were above 1–3 µM (Lohman et al. 2016).

### Other HDAC inhibitors

Emodin, a compound extracted from the root of *Rheum palmatum L.,* showed potential as an anti-proliferative agent on synoviocytes from individuals with RA. A study done by Ha and colleagues showed that under a hypoxic environment, the presence of emodin significantly reduces the proliferation of RA synoviocytes stimulated by factors such as LPS and IL-1 beta (IL-1b). Administering emodin notably decreased the synthesis of pro-inflammatory cytokines, including TNF-α, IL-6, and IL-8. Additionally, emodin exhibited inhibitory effects on the synthesis of mediators such as prostaglandin E2 (PGE2), MMP-1, and MMP-13. Furthermore, emodin consistently reduced the levels of the commonly seen and studied biomarker for angiogenesis, which is the VEGF (Ha et al. 2011). Largazole (LAR) is a marine-derived class I HDACi. A study by Ahmed et al., in 2013 shows that LAR dose dependently increases the production of TNF-α + LAR-induced CAMs while simultaneously suppressing MMP-2 activity. Notably, adding Tubastatin A, a selective inhibitor of HDAC6, to LAR,reduced ICAM-1 and VCAM-1 expression induced by TNF-α and LAR. Moreover, the activity of MMP-2 was entirely blocked by this combination treatment (Ahmed et al. 2013).

Another HDACi is NK-HDAC1, which shows promising results and displayed properties such as good bioavailability and potency (Hou et al. 2012). This implicated that NK-HDAC-1 may become a promising therapeutic agent for RA treatment. Li et al., in their 2013 study, noted the potential of NK-HDAC-1, an HDAC inhibitor, as a therapeutic avenue for CIA and pathogenic FLSs obtained from individuals affected by RA. Notably, programmed cell death or apoptosis was induced in RA FLSs upon NK-HDAC-1. Additionally, upon cell cycle analysis, it was observed that treatment with NK-HDAC-1 resulted in a cell cycle halt in the G2/M phase, thereby suppressing the proliferation of FLS. These findings offer insights into the potential therapeutic benefits of NK-HDAC-1 in the context of RA. NK-HDAC-1 subjection, a modest reduction was observed in the MMP-9 expression levels (Li et al. 2013). Butyrate's therapeutic properties were studied in an autoimmune arthritis animal model, showing lower arthritis scores and incidence. It inhibited HDAC2 and HDAC8 in osteoclasts and T-cells, controlled TH17/Treg cell balance and inhibited osteoclastogenesis. However, it did not affect inflammatory arthritis in IL-10-knockkout mice (Kim et al. 2018). The novel HDAC6 inhibitor CKD-506 dampens inflammatory reactions by monocytes/macrophages, enhances Treg activity, and mitigates arthritis severity in a mouse model of RA. Consequently, CKD-506 presents a potential novel and efficient therapeutic approach for RA (Park et al.., 2020). The combined therapy of M-134 and Tofactinib demonstrated potent synergistic effects, evident through improvements in clinical scores and histological alterations, while avoiding adverse effects like thymus weight loss and elevated liver enzymes. This combination could help therapeutic efficacy in treating RA suggests a potentially efficient approach for managing the condition (Bae et al. 2021).

**Fig. 4:**
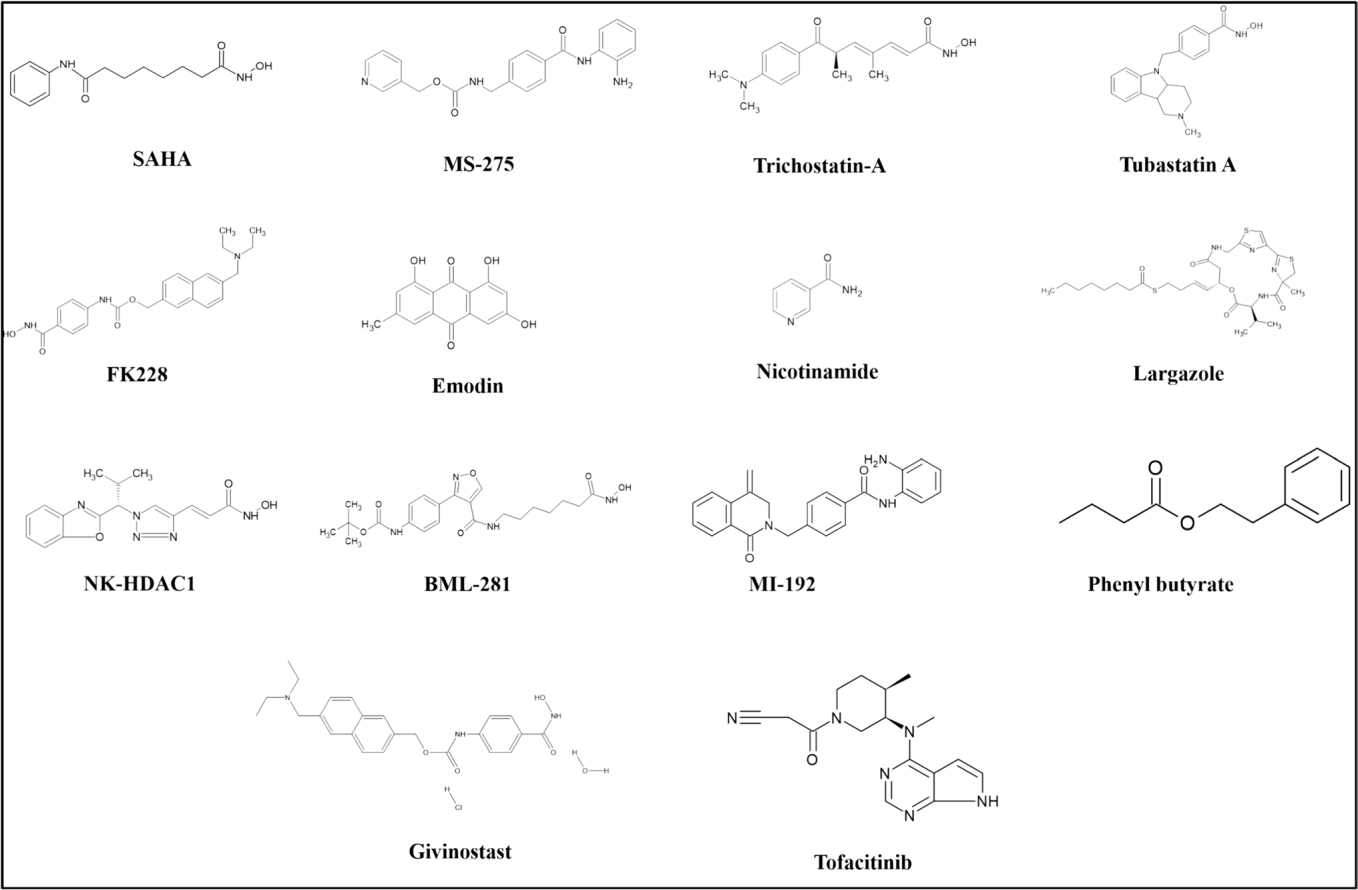
Chemical structures of HDAC inhibitors

HDACi can reduce inflammation and disease in RA patients by stopping synovial fibroblast proliferation. It inhibits p21 cell cycle inhibition and induces apoptosis in RA FLS. Reservatrol and SIRT6 can also reduce pro-inflammatory cytokines and chemokines. HDAC2 is elevated in RA and CIA rats, leading to joint swelling and increased RA. Silencing HDAC2 blocks chemokine ligand 7 (CCL7) expression, reducing FLS invasion and inflammatory factors (Mao et al. 2023). (Table 1; Fig. 4).

**Table 1:** HDAC inhibitors, targets of HDAC and pathological/histological effects of on RA

HDAC inhibitor	Affecting protein/transcription factor	Pathological/histological effects	Reference
Phenyl butyrate and TSA	Triggered p21^Cip1^ and p16^INK4^ expression in synovial cells	Molecular targets mediated, disease progression is alleviated. Lesser infiltration of subintimal mononuclear cells	Chung et al. 2003
FK228	Reduced levels of IL-1 and TNF-α, Elevated expression of p21^Cip1^ and p16^INK4a^	FK228 modulated cell cycle, and cell cycle arrest induced	Nishida et al. 2004
TSA	Enhanced expression of p21^WAF1/CIP1^, aided the Fas-induced pathway	Anti-rheumatic effects exhibited by TSA, RASF cell growth inhibited, cell death and apoptosis induced	Morinobu et al. 2006
FK228	Lowered expression of VEGF and HIF-1	Down-regulation of pro-angiogenic factors, within synovial tissue, angiogenesis prevented	Manabe et al 2008
MS-275 and SAHA	P21 induced, VEGF, MMP-2 and MMP-9 downregulated, reduced the nuclear accumulation of NF-κB p65 and the secretion of IL-6, IL-18, NO	Anti-rheumatic effects exhibited, disease alleviated. Cell cycle arrest induced in the G0/G1 phase	Choo et al. 2010
TSA and NIC	IL-8 production inhibited by 30%, IL-6 by 70%, Bfl-1 expression decreased	Disease Intensity reduced. Apoptosis induced in FLSs and macrophages	Grabiec et al. 2010
Emodin	Decreased production of proinflammatory cytokines such as TNF-α, IL-6, and IL-8, Inhibited synthesis of PGE2, MMPs like MMP-1, MMP-13, Reduced VEGF levels. Reduced expression of genes such as COX-2, HIF-1α	Inhibited Inflammation and decreases RA severity. RASF proliferation stimulated by IL-1 beta (IL-1b) reduced	Ha et al. 2011
TSA and Givinostat	Accumulation IL-6 mRNA suppressed, and degradation induced. Inflammatory cytokine mRNA modulated as a mechanism	Showed positive effects of targeting HDAC activity and suppressed RA pathogenesis. Viability of RASF unaffected	Grabiec et al., 2012
MI192 and TSA Individually	At high doses, suppresses TNF production. 10 μM to 5 nM, inhibits IL-6 in a dose-dependant manner	RA severity was alleviated. Increased HDAC activity and cytokine production suppressed in RA PBMCs	Gillespie et al. 2012
LAR and TSA in combination	Reduced TNF-α induced ICAM-1 and VCAM-1 expression, MMP-2 Activity completely blocked. LAR inhibited HDAC1 and HDAC5 expression in RASFs	Aided in RA decrease	Ahmed et al. 2013
MS-275 and SAHA	Several Inflammatory mediators such as NO, IL-1β, IL-6, IL-18 and TNF-α were supressed	p38 MAPK signaling pathway suppressed. Anti-Inflammatory effects exhibited, RA alleviated	Choo et al. 2013
Tubastatin	Inhibited synthesis of proinflammatory cytokines including TNF-α and IL-6. Secretion of proinflammatory chemokines such as NO reduced. Resulted in a 59% reduction in IL-6 levels in paw tissues	In mouse arthritis models, resulted in a significant reduction in paw volume	Vishwakarma et al. 2013
NK-HDAC-1	Notable reduction in the production of proinflammatory cytokines such as IL-6 and the expression of MMP-3	Proliferation and viability of RA-FLS suppressed. G2/M phase cell cycle arrest induced	Li et al. 2013
SAHA and BML281	Upto doses of 30 µM, production of cytokines IL12p40 and IL6 was suppressed. Concentrations above 1-3 µM increased production of TNF -α and IL-1 in a dose-dependent manner	Both treatments showed positive effects at lower dosages (1 mg kg^−1^ day^−1^) and showed disease-aggravating effects at higher doses (5 mg kg^−1^ day^−1^). Both reduced numbers of ED1 + immune cells in comparison to controls, At low doses, reduced pathogenic osteoclast and macrophage numbers	Lohman et al. 2016
MS-275	Reduction in TNF production in affected joints in CIA rat models	Mediated tissue damage. Reduced extent of bone and cartilag degradation	Hawtree et al. 2015
TSA	Inhibited upregulation of MMP-2 and MMP-9, Suppressed the PI3K/Akt signalling pathway, Enhanced E-Cadherin expression	Exhibited antiinvasive and anti-survival activity. RA FLS invading capacity decreases, viability decreased, concentration-dependant apoptosis induced,	Zhang and Zhang 2016
CKD-L compared to Tubastatin A and Givinostat	In response to CDK-L treatment, secretion of TNF, IL-1 blocked, secretion of IL-10 elevated, TNF mRNA levels reduced. TSA and ITF2357 decreased TSA secretion	Reduced the arthritis score in mice models. Teff cell proliferation dose-dependently decreased after treatment with CKD-L and TSA. ITF2357 decreased viability at concentrations over one µM	Oh et al. 2017
Givinostat	Stability of mRNA transcripts of IL-6, IL-8, CXCL2, PTGS2 decreased, and expression was post-translationally altered. Inhibition of phosphorylation and subsequent inactivation of TTP, post-transcriptionally	Absence of TTP results in a pronounced inflammatory response in mice models	Angiolilli et al. 2018
M-134 and Tofactinib	Tofactinib decreased TNF -α levels. M-134 decreased expression of IL-17A, IP-10 and TNF -α in a dose-dependent manner. Combined treatment resulted in a more severe decrease in IL-8 and IL-10 levels as compared to when they were administered individually	Combination therapy regulated cytokines, chemokines and CAMs, and served as a novel therapeutic avenue	Bae et al., 2020
CDK-506 (M-134)	Reduced TNF -α and IL-6 production in a dose-dependent manner. Production of MMP-1 and MMP-6, IL-8 and chemokines like CCL2 and CXCL10 also decreased	Ameliorated RA severity and suppresses the body’s inflammatory response. Reduced T-cell proliferation, reduced inflammation	Park et al. 2020

## Conclusion and future prospective

Studies have specifically focused on the impact of Class I HDAC inhibition, recognizing their involvement in modulating key pro-inflammatory cytokines and pathways associated with RA pathogenesis. Further investigation into HDAC isoforms is required to assess their specific mechanisms in the context of RA. The exploration of HDACis as a therapeutic avenue for RA represents an exciting frontier in the field of autoimmune disease treatment. As research progresses, the potential for personalized, targeted therapies holds great promise for improving outcomes and quality of life for RA patients. The journey from bench to bedside may still be in its early stages, but the strides made so far suggest that HDACis could be a transformative force in the evolving landscape of RA.

Though the promising potential benefits of HDACis in treating RA are acknowledged, there are persistent challenges. These include determining the most effective dosages, managing potential side effects, and ensuring long-term safety. Ongoing research efforts focus on understanding the precise mechanisms of action. Additionally, understanding the specific mechanisms of action and identifying the for HDAC inhibitor therapy are critical for successful integration into standard RA management.

## Data Availability

No additional data are available.

## Abbreviations

CAM: Cell adhesion molecule
CCL7: Chemokine ligand 7
CCR6: Chemokine receptor 6
CIA: Collagen-induced arthritis
FLS: Fibroblast-like synoviocytes
HAT: Histone acetyltransferase
HDAC: Histone deacetylase
HDACi: Histone deacetylase inhibitor
HIF: Hypoxia-inducible factor
ICAM: Intercellular adhesion molecule
IFN: Interferon
IL: Interleukin
ILD: Interstitial lung disease
LPS: Lipopolysaccharide
MMP: Matrix metalloproteinase
mRNA: Messenger RNA
NF-κB: Nuclear factor kappa B
NIC: Nicotinamide
OA: Osteoarthritis
PBMC: Peripheral blood mononuclear cells
RA: Rheumatoid arthritis
RA-ILD: Rheumatoid arthritis-associated interstitial lung disease
RASF: Rheumatoid arthritis synovial fibroblasts
SAHA: Suberoylanilide hydroxamic acid
TNF: Tumor-necrosis factor
TSA: Trichostatin A
VCAM: Vascular cell adhesion molecule
VEGF: Vascular endothelial growth factor

## References

[CR1] Ahmed S, Riegsecker S, Beamer M, Rahman A, Bellini JV, Bhansali P, Tillekeratne LV (2013) Largazole, a class I histone deacetylase inhibitor, enhances TNF-α-induced ICAM-1 and VCAM-1 expression in rheumatoid arthritis synovial fibroblasts. Toxicol. 10.1016/j.taap.2013.04.014 10.1016/j.taap.2013.04.014 PMC3766723 23632129

[CR2] Angiolilli C, Grabiec AM, Ferguson BS, Ospelt C, Fernandez BM, van Es IE, Reedquist KA (2014) Inflammatory cytokines epigenetically regulate rheumatoid arthritis fibroblast-like synoviocyte activation by suppressing HDAC5 expression. Ann Rheum Dis. 10.1136/annrheumdis-2014-205635 25452308 10.1136/annrheumdis-2014-205635 PMC5336378

[CR3] Angiolilli C, Kabala PA, Grabiec AM, Van Baarsen IM, Ferguson BS, García S, Reedquist KA (2017) Histone deacetylase 3 regulates the inflammatory gene expression programme of rheumatoid arthritis fibroblast-like synoviocytes. Ann Rheum Dis. 10.1136/annrheumdis-2015-209064 27457515 10.1136/annrheumdis-2015-209064 PMC5264225

[CR4] Angiolilli C, Kabala PA, Grabiec AM, Rossato M, Lai WS, Fossati G, Radstake TR (2018) Control of cytokine mRNA degradation by the histone deacetylase inhibitor ITF2357 in rheumatoid arthritis fibroblast-like synoviocytes: beyond transcriptional regulation. Arthritis Res Ther. 10.1186/s13075-018-1638-4 30029685 10.1186/s13075-018-1638-4 PMC6053802

[CR5] Bae D, Choi Y, Lee J, Ha N, Suh D, Baek J, Son W (2021) M-134, a novel HDAC6-selective inhibitor, markedly improved arthritic severity in a rodent model of rheumatoid arthritis when combined with tofacitinib. Pharmacological Rep. 10.1007/s43440-020-00188-x 10.1007/s43440-020-00188-x 33188511

[CR6] Burrage PS, Mix KS, Brinckerhoff CE (2006) Matrix metalloproteinases: role i arthritis. Front Biosci. 10.2741/1817 16146751 10.2741/1817

[CR7] Choi J, Gang S, Ramalingam M, Hwang J, Jeong H, Yoo J, Jang S (2023) BML-281 promotes neuronal differentiation by modulating Wnt/Ca2+ and Wnt/PCP signaling pathway. Mol Cell Biochem. 10.1007/s11010-023-04857-2 37768498 10.1007/s11010-023-04857-2

[CR8] Choo QY, Ho PC, Tanaka Y, Lin HS (2010) Histone deacetylase inhibitors MS-275 and SAHA induced growth arrest and suppressed lipopolysaccharide-stimulated NF-κB p65 nuclear accumulation in human rheumatoid arthritis synovial fibroblastic E11 cells. Rheumatology. 10.1093/rheumatology/keq108 20421217 10.1093/rheumatology/keq108

[CR9] Choo QY, Ho PC, Tanaka Y, Lin HS (2013) The histone deacetylase inhibitors MS-275 and SAHA suppress the p38 mitogen-activated protein kinase signaling pathway and chemotaxis in rheumatoid arthritic synovial fibroblastic E11 cells. Molecules 18:14085 24241152 10.3390/molecules181114085 PMC6270078

[CR10] Chung YL, Lee MY, Wang AJ, Yao LF (2003) A therapeutic strategy uses histone deacetylase inhibitors to modulate the expression of genes involved in the pathogenesis of rheumatoid arthritis. Mol Ther. 10.1016/s1525-0016(03)00235-1 14599803 10.1016/s1525-0016(03)00235-1

[CR11] Costenbader KH, Gay S, Alarcón-Riquelme ME, Iaccarino L, Doria A (2012) Genes, epigenetic regulation and environmental factors: which is the most relevant in developing autoimmune diseases? Autoimmun Rev. 10.1016/j.autrev.2011.10.022 22041580 10.1016/j.autrev.2011.10.022

[CR12] Cui SN, Chen ZY, Yang XB, Chen L, Yang YY, Pan SW, Shang Y (2019) Trichostatin A modulates the macrophage phenotype by enhancing autophagy to reduce inflammation during polymicrobial sepsis. Int Immunopharmcol. 10.1016/j.intimp.2019.105973 10.1016/j.intimp.2019.105973 31677992

[CR13] Elshabrawy HA, Chen Z, Volin MV, Ravella S, Virupannavar S, Shahrara S (2015) The pathogenic role of angiogenesis in rheumatoid arthritis. Angiogenesis 18:433 26198292 10.1007/s10456-015-9477-2 PMC4879881

[CR14] Furlan A, Monzani V, Reznikov LL, Leoni F, Fossati G, Modena D, Dinarello CA (2011) Pharmacokinetics, safety and inducible cytokine responses during a phase 1 trial of the oral histone deacetylase inhibitor ITF2357 (givinostat). Mol Med. 10.2119/molmed.2011.00020 21365126 10.2119/molmed.2011.00020 PMC3105139

[CR15] Gerosa M, De Angelis V, Riboldi P, Meroni PL (2008) Rheumatoid arthritis: a female challenge. WHNP. 10.2217/17455057.4.2.195 10.2217/17455057.4.2.195 19072521

[CR16] Gillespie J, Savic S, Wong C, Hempshall A, Inman M, Emery P, McDermott MF (2012) Histone deacetylases are dysregulated in rheumatoid arthritis and a novel histone deacetylase 3–selective inhibitor reduces interleukin-6 production by peripheral blood mononuclear cells from rheumatoid arthritis patients. Arthritis Rheum. 10.1002/art.33382 21952924 10.1002/art.33382

[CR17] Göschl L, Preglej T, Boucheron N, Saferding V, Müller L, Platzer A, Bonelli M (2020) Histone deacetylase 1 (HDAC1): a key player of T cell-mediated arthritis. J Autoimmun. 10.1016/j.jaut.2019.102379 31883829 10.1016/j.jaut.2019.102379

[CR18] Grabiec AM, Reedquist KA (2013) The ascent of acetylation in the epigenetics of rheumatoid arthritis. Nat Rev Rheumatol. 10.1038/nrrheum.2013.17 23439035 10.1038/nrrheum.2013.17

[CR19] Grabiec AM, Krausz S, De Jager W, Burakowski T, Groot D, Sanders ME, Reedquist KA (2010) Histone deacetylase inhibitors suppress inflammatory activation of rheumatoid arthritis patient synovial macrophages and tissue. J Immunol. 10.4049/jimmunol.0901467 20100935 10.4049/jimmunol.0901467

[CR20] Grabiec AM, Korchynskyi O, Tak PP, Reedquist KA (2011) Histone deacetylase inhibitors suppress rheumatoid arthritis fibroblast-like synoviocyte and macrophage IL-6 production by accelerating mRNA decay. Ann Rheum Dis. 10.1136/ard.2011.154211 21953341 10.1136/ard.2011.154211 PMC3277722

[CR21] Ha MK, Song YH, Jeong SJ, Lee HJ, Jung JH, Kim B, Kim SH (2011) Emodin inhibits proinflammatory responses and inactivates histone deacetylase 1 in hypoxic rheumatoid synoviocytes. Biol Pharm Bull. 10.1248/bpb.34.1432 21881229 10.1248/bpb.34.1432

[CR22] Hawtree S, Muthana M, Wilson AG (2013) The role of histone deacetylases in rheumatoid arthritis fibroblast-like synoviocytes. Biochem Soc Trans. 10.1042/BST20130053 23697938 10.1042/BST20130053

[CR23] Hawtree S, Muthana M, Wilkinson JM, Akil M, Wilson AG (2015) Histone deacetylase 1 regulates tissue destruction in rheumatoid arthritis. Hum Mol Genet. 10.1093/hmg/ddv258 26152200 10.1093/hmg/ddv258

[CR24] Horiuchi M, Morinobu A, Chin T, Sakai Y, Kurosaka M, Kumagai S (2009) Expression and function of histone deacetylases in rheumatoid arthritis synovial fibroblasts. J Rheumatol. 10.3899/jrheum.081115 19531758 10.3899/jrheum.081115

[CR25] Hou J, Li Z, Fang Q, Feng C, Zhang H, Guo W, Wang PG (2012) Discovery and extensive in vitro evaluations of NK-HDAC-1: a chiral histone deacetylase inhibitor as a promising lead. J Med Chem. 10.1021/jm201496g 22435669 10.1021/jm201496g

[CR26] Huber LC, Brock M, Hemmatazad H, Giger OT, Moritz F, Trenkmann M, Jüngel A (2007) Histone deacetylase/acetylase activity in total synovial tissue derived from rheumatoid arthritis and osteoarthritis patients. Arthritis Rheum. 10.1002/art.22512 17393417 10.1002/art.22512

[CR27] Ito T, Ouchida M, Morimoto Y, Yoshida A, Jitsumori Y, Ozaki T, Shimizu K (2005) Significant growth suppression of synovial sarcomas by the histone deacetylase inhibitor FK228 in vitro and in vivo. Cancer Let. 10.1016/j.canlet.2004.10.030 10.1016/j.canlet.2004.10.030 15914281

[CR28] Kawabata T, Nishida K, Takasugi K, Ogawa H, Sada K, Kadota Y, Makino H (2010) Increased activity and expression of histone deacetylase 1 in relation to tumor necrosis factor-alpha in synovial tissue of rheumatoid arthritis. Arthritis Res Ther 12:R133 20609223 10.1186/ar3071 PMC2945023

[CR29] Kim DS, Kwon JE, Lee SH, Kim EK, Ryu JG, Jung KA, Kwok SK (2018) Attenuation of rheumatoid inflammation by sodium butyrate through reciprocal targeting of HDAC2 in osteoclasts and HDAC8 in T cells. Front Immunol. 10.3389/fimmu.2018.01525 30034392 10.3389/fimmu.2018.01525 PMC6043689

[CR30] Klein K, Gay S (2013) Epigenetic modifications in rheumatoid arthritis, a review. Curr Opin Pharmacol. 10.1016/j.coph.2013.01.007 23384968 10.1016/j.coph.2013.01.007

[CR31] Kong S, Yeung P, Fang D (2013) The class III histone deacetylase sirtuin 1 in immune suppression and its therapeutic potential in rheumatoid arthritis. J Genet Genomics. 10.1016/j.jgg.2013.04.001 23876775 10.1016/j.jgg.2013.04.001 PMC4007159

[CR32] Konstantinopoulos PA, Vandoros GP, Papavassiliou AG (2006) FK228 (depsipeptide): a HDAC inhibitor with pleiotropic antitumor activities. Cancer Chemother Pharmacol. 10.1007/s00280-005-0182-5 16435156 10.1007/s00280-005-0182-5

[CR33] Lee DM, Weinblatt ME (2001) Rheumatoid arthritis. Lancet. 10.1016/S0140-6736(01)06075-511784652

[CR34] Lee EC, Kim YM, Lim HM, Ki GE, Seo YK (2020) The histone deacetylase inhibitor (MS-275) promotes differentiation of human dental pulp stem cells into odontoblast-like cells independent of the MAPK signaling system. Int J Mol Sci. 10.3390/ijms21165771 32796747 10.3390/ijms21165771 PMC7460873

[CR35] Li M, Liu X, Sun X, Wang Z, Guo W, Hu F, Li Z (2013) Therapeutic effects of NK-HDAC-1, a novel histone deacetylase inhibitor, on collagen-induced arthritis through the induction of apoptosis of fibroblast-like synoviocytes. Inflammation. 10.1007/s10753-013-9616-0 23549599 10.1007/s10753-013-9616-0

[CR36] Li Y, Zhou M, Lv X, Song L, Zhang D, He Y, Wang D (2018) Reduced activity of HDAC3 and increased acetylation of histones H3 in peripheral blood mononuclear cells of patients with rheumatoid arthritis. J Immunol Res 2018:1 10.1155/2018/7313515 PMC6192092 30402512

[CR37] Lin HS, Hu CY, Chan HY, Liew YY, Huang HP, Lepescheux L, Clément-Lacroix P (2007) Anti-rheumatic activities of histone deacetylase (HDAC) inhibitors in vivo in collagen-induced arthritis in rodents. Br J Pharmacol. 10.1038/sj.bjp.0707165 17325656 10.1038/sj.bjp.0707165 PMC2013883

[CR38] Lohman RJ, Iyer A, Fairlie TJ, Cotterell A, Gupta P, Reid RC, Fairlie DP (2016) Differential anti-inflammatory activity of HDAC inhibitors in human macrophages and rat arthritis. J Pharmacol Exp Ther. 10.1124/jpet.115.229328 26660228 10.1124/jpet.115.229328

[CR39] Lv X, Qiu J, Hao T, Zhang H, Jiang H, Tan Y (2021) HDAC inhibitor Trichostatin A suppresses adipogenesis in 3T3-L1 preadipocytes. Aging 13:17489 34232916 10.18632/aging.203238 PMC8312440

[CR40] Manabe H, Nasu Y, Komiyama T, Furumatsu T, Kitamura A, Miyazawa S, Nishida K (2008) Inhibition of histone deacetylase down-regulates the expression of hypoxia-induced vascular endothelial growth factor by rheumatoid synovial fibroblasts. Inflamm Res. 10.1007/s00011-007-7036-z 18209959 10.1007/s00011-007-7036-z

[CR41] Mao D, Jiang H, Zhang F, Yang H, Fang X, Zhang Q, Zhao G (2023) HDAC2 exacerbates rheumatoid arthritis progression via the IL-17-CCL7 signaling pathway. Environ Toxicol. 10.1002/tox.23802 37021908 10.1002/tox.23802

[CR42] Mizutani H, Hiraku Y, Tada-Oikawa S, Murata M, Ikemura K, Iwamoto T, Kawanishi S (2010) Romidepsin (FK228), a potent histone deacetylase inhibitor, induces apoptosis through the generation of hydrogen peroxide. Cancer Sci. 10.1111/j.1349-7006.2010.01645.x 20624163 10.1111/j.1349-7006.2010.01645.x PMC11159834

[CR43] Morinobu A, Wang B, Liu J, Yoshiya S, Kurosaka M, Kumagai S (2006) Trichostatin A cooperates with Fas-mediated signal to induce apoptosis in rheumatoid arthritis synovial fibroblasts. J Rheumatol 33:1052–1060 16755652

[CR44] Mulherin D, Fitzgerald O, Bresnihan B (1996) Synovial tissue macrophage populations and articular damage in rheumatoid arthritis. Arthritis Rheumatol. 10.1002/art.1780390116 10.1002/art.1780390116 8546720

[CR45] Nakamura C, Matsushita I, Kosaka E, Kondo T, Kimura T (2008) Anti-arthritic effects of combined treatment with histone deacetylase inhibitor and low-intensity ultrasound in the presence of microbubbles in human rheumatoid synovial cells. Rheumatology. 10.1093/rheumatology/ken003 18281366 10.1093/rheumatology/ken003

[CR46] Nishida K, Komiyama T, Miyazawa SI, Shen ZN, Furumatsu T, Doi H, Asahara H (2004) Histone deacetylase inhibitor suppression of autoantibody-mediated arthritis in mice via regulation of p16INK4a and p21WAF1/Cip1 expression. Arthritis Rheumatol. 10.1002/art.20709 10.1002/art.20709 15476220

[CR47] O’Dell JR (2004) Therapeutic strategies for rheumatoid arthritis. N Engl J Med. 10.1056/nejmra040226 15201416 10.1056/NEJMra040226

[CR48] Oh BR, Suh DH, Bae D, H N, Choi YI, Yoo HJ, Song YW, (2017) Therapeutic effect of a novel histone deacetylase 6 inhibitor, CKD-L, on collagen-induced arthritis in vivo and regulatory T cells in rheumatoid arthritis in vitro. Arthritis Res Ther. 10.1186/s13075-017-1357-2 28673326 10.1186/s13075-017-1357-2 PMC5496370

[CR49] Park JK, Jang YJ, Oh BR, Shin J, Bae D, Ha N, Song YW (2020) Therapeutic potential of CKD-506, a novel selective histone deacetylase 6 inhibitor, in a murine model of rheumatoid arthritis. Arthritis Res Ther. 10.1186/s13075-020-02258-0 32711562 10.1186/s13075-020-02258-0 PMC7382061

[CR50] Ruijter AJD, Gennip AHV, Caron HN, Kemp S, Kuilenburg ABV (2003) Histone deacetylases (HDACs): characterization of the classical HDAC family. Biochem J. 10.1042/bj20021321 12429021 10.1042/BJ20021321 PMC1223209

[CR51] Savino AM, Sarno J, Trentin L, Vieri M, Fazio G, Bardini M, Cazzaniga G (2017) The histone deacetylase inhibitor givinostat (ITF2357) exhibits potent anti-tumor activity against CRLF2-rearranged BCP-ALL. Leukemia. 10.1038/leu.2017.93 28331226 10.1038/leu.2017.93

[CR52] Shen S, Svoboda M, Zhang G, Cavasin MA, Motlova L, McKinsey TA, Kozikowski AP (2020) Structural and in vivo characterization of Tubastatin A, a widely used histone deacetylase 6 inhibitor. ACS Med Chem Lett 11:706 32435374 10.1021/acsmedchemlett.9b00560 PMC7236036

[CR53] Smith DA, Germolec DR (1999) Introduction to immunology and autoimmunity. Environ. 10.1289/ehp.99107s5661 10.1289/ehp.99107s5661 PMC1566249 10502528

[CR54] Sweeney SE, Firestein GS (2004) Rheumatoid arthritis: regulation of synovial inflammation. Int J Biochem Cell Bio. 10.1016/s1357-2725(03)00259-0 10.1016/s1357-2725(03)00259-0 14687914

[CR55] Thalhamer T, McGrath MA, Harnett MM (2008) MAPKs and their relevance to arthritis and inflammation. Rheumatology. 10.1093/rheumatology/kem297 18187523 10.1093/rheumatology/kem297

[CR56] Toussirot E, Abbas W, Khan KA, Tissot M, Jeudy A, Baud L, Herbein G (2013) Imbalance between HAT and HDAC activities in the PBMCs of patients with ankylosing spondylitis or rheumatoid arthritis and influence of HDAC inhibitors on TNF alpha production. PLoS ONE 8:e70939 24039666 10.1371/journal.pone.0070939 PMC3748901

[CR57] Vijaykrishnaraj M, Patil P, Ghate SD, Bhandary AK, Haridas VM, Shetty P (2023) Efficacy of HDAC inhibitors and epigenetic modulation in the amelioration of synovial inflammation, cellular invasion, and bone erosion in rheumatoid arthritis pathogenesis. Int Immonopharmacol. 10.1016/j.intimp.2023.110644 10.1016/j.intimp.2023.110644 37454631

[CR58] Vishwakarma S, Iyer LR, Muley M, Singh PK, Shastry A, Saxena A, Narayanan S (2013) Tubastatin, a selective histone deacetylase 6 inhibitor shows anti-inflammatory and anti-rheumatic effects. Int Immunopharmacol. 10.1016/j.intimp.2013.03.016 23541634 10.1016/j.intimp.2013.03.016

[CR59] Vojinovic J, Damjanov N (2011) HDAC inhibition in rheumatoid arthritis and juvenile idiopathic arthritis. Mol Med. 10.2119/molmed.2011.00030 21308151 10.2119/molmed.2011.00030 PMC3105145

[CR60] Wu D, Luo Y, Li T, Zhao X, Lv T, Fang G, Pang Y (2022) Systemic complications of rheumatoid arthritis: focus on pathogenesis and treatment. Front Immunol. 10.3389/fimmu.2022.1051082 36618407 10.3389/fimmu.2022.1051082 PMC9817137

[CR61] Yuan H, Jiao L, Yu N, Duan H, Yu Y, Bai Y (2020) Histone deacetylase 3-mediated inhibition of microRNA-19a-3p facilitates the development of rheumatoid arthritis-associated interstitial lung disease. Front Physiol. 10.3389/fphys.2020.549656 33343379 10.3389/fphys.2020.549656 PMC7746846

[CR62] Zhang Y, Zhang B (2016) Trichostatin A, an inhibitor of histone deacetylase, inhibits the viability and invasiveness of hypoxic rheumatoid arthritis fibroblast-like synoviocytes via PI3K/Akt signaling. J Biochem Mol Toxicol. 10.1002/jbt.21774 26509796 10.1002/jbt.21774

